# Cross talk between mesenchymal and glioblastoma stem cells: Communication beyond controversies

**DOI:** 10.1002/sctm.20-0161

**Published:** 2020-06-15

**Authors:** Adriana Bajetto, Stefano Thellung, Irene Dellacasagrande, Aldo Pagano, Federica Barbieri, Tullio Florio

**Affiliations:** ^1^ Dipartimento di Medicina Interna Università di Genova Genova Italy; ^2^ Dipartimento di Medicina Sperimentale Università di Genova Genova Italy; ^3^ IRCCS Ospedale Policlinico San Martino Genova Italy

**Keywords:** cancer stem cells, extracellular vesicles, glioblastoma, mesenchymal stem cells, secretoma

## Abstract

Mesenchymal stem cells (MSCs) can be isolated from bone marrow or other adult tissues (adipose tissue, dental pulp, amniotic fluid, and umbilical cord). In vitro, MSCs grow as adherent cells, display fibroblast‐like morphology, and self‐renew, undergoing specific mesodermal differentiation. High heterogeneity of MSCs from different origin, and differences in preparation techniques, make difficult to uniform their functional properties for therapeutic purposes. Immunomodulatory, migratory, and differentiation ability, fueled clinical MSC application in regenerative medicine, whereas beneficial effects are currently mainly ascribed to their secretome and extracellular vesicles. MSC translational potential in cancer therapy exploits putative anti‐tumor activity and inherent tropism toward tumor sites to deliver cytotoxic drugs. However, controversial results emerged evaluating either the therapeutic potential or homing efficiency of MSCs, as both antitumor and protumor effects were reported. Glioblastoma (GBM) is the most malignant brain tumor and its development and aggressive nature is sustained by cancer stem cells (CSCs) and the identification of effective therapeutic is required. MSC dualistic action, tumor‐promoting or tumor‐targeting, is dependent on secreted factors and extracellular vesicles driving a complex cross talk between MSCs and GBM CSCs. Tumor‐tropic ability of MSCs, besides providing an alternative therapeutic approach, could represent a tool to understand the biology of GBM CSCs and related paracrine mechanisms, underpinning MSC‐GBM interactions. In this review, recent findings on the complex nature of MSCs will be highlighted, focusing on their elusive impact on GBM progression and aggressiveness by direct cell‐cell interaction and via secretome, also facing the perspectives and challenges in treatment strategies.

## Graphical abstract

Diagrammatic representation of the interactions between mesenchymal stem cells and glioblastoma cells, involving also the other nonmalignant stromal and immune cells within the tumor microenvironment. In the figure are highlighted the critical pathways that may support or impair tumor growth via a variety of mechanisms

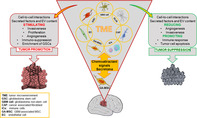

**Significance statement**

Mesenchymal stem cells (MSCs) attract interest for their unique potential properties, which make them a suitable resource for the treatment of several human disorders. As yet, MSC‐based therapy has been applied to degenerative and inflammatory diseases, tissue repair, and to fight cancer. The present review focuses on recent findings from preclinical studies on MSCs in oncology as a source of soluble factors and extracellular vesicles (EVs), underscoring MSC interaction with glioblastoma (GBM) in in vivo and in vitro models. Importantly, because MSCs may promote or suppress tumor growth, they act as a double‐edged sword in the GBM model, as in other tumor types. The review also addresses the evidence for challenges, risks, and further research investigations needed to carefully explore and define the actual MSC nature, before their clinical translation as effective and safe tools for future anticancer approaches.

## 1 INTRODUCTION

### 1.1 Mesenchymal stem cells: Origin and isolation

Mesenchymal stem cells (MSCs) are adult multipotent stem cells that harbor, although rare, in the bone marrow (BM) and in almost all body tissues.^1^, ^2^ The classical and widely used sources of human MSCs for clinical settings are the BM, the adipose tissue (AT),^3^ and the umbilical cord (UC),^4^ exhibiting peculiar in vivo biology and different native functions (see Table 1 for details), which represent a promising tool for cell therapy, tissue engineering, and regenerative medicine. More recently, Wharton's jelly (WJ), amnion and corion,^5^ and umbilical cord blood (UCB)^6^ were proposed as alternative source of MSCs, although in the UCB they were identified at very low frequency as compared to other tissues.^7^ MSCs have been harvested also from endometrium, synovium,^8^ muscle,^9^ skin, placental,^10^ and dental pulp.^11^ Increasing evidence proposes the use of MSCs as promising therapeutic approach for the treatment of several diseases and applications in the fields of regenerative medicine, neuroscience, oncology, pharmacology, and bioengineering. Currently, more than 500 studies (recruiting, not yet recruiting, active not recruiting on May 2020; search terms: MSC and mesenchymal stromal cell) are registered for MSCs, according to https://ClinicalTrials.gov.

**TABLE 1:** In vivo and in vitro biological characteristics of MSCs most commonly used in clinical studies

MSC source	In vivo role		In vitro biological features		
		Collection/isolation	Level of differentiation ability	Immunophenotype^18^, ^19^ (beyond ISCT minimal criteria, main markers)	Proliferation/senescence
Bone marrow	Formation and maintenance of the hematopoietic stem cell niche	Invasive collection procedure/0.001%‐0.01% of the total BM nucleated cells	High estrogenic and chondrogenic potential	Stro‐1^+^, SSEA‐4^+^, CD146^+^, CD106^+,^ CD271^+^	Low proliferative capacity and clonogenicity/senescence after ∼12 in vitro passages
Adipose tissue	Localized within the stromal vascular fraction regulate local of angiogenesis and vessel remodeling	Ease of collection/high availability (∼500‐fold as compared to BM‐MSC)	High adipogenic potential, endothelial cells	CD34^+^ (at least in early in vitro passages), CD10^+^, CD36^+^, CD49d^+^, CD106^−^	Good proliferative capacity and high clonogenicity senescence
Umbilical cord	Maintenance of stromal tissue by differentiating into myofibroblasts to elaborate ECM	Non‐invasive collection procedure/low frequency of MSC	High chondrogenic potential	Stro‐1^−^, SSEA‐4^−^, CD146^+^, CD271^−^	High proliferative capacity and clonogenicity (compared to BM and AT)/low senescence

At present, the extension of the concept and term MSC, originally circumscribed to nonhematopoietic BM‐derived cells, to cells derived from additional postnatal tissues, rises some concerns. In particular, the MSC concept was questioned as far as two main defining stem cell assumptions, self‐renewal and multipotency, and concerning the experimental approaches used for isolation and characterization. Indeed, MSCs from different tissue sources comprise differences in cell populations, which display distinct characteristics, technical difficulties and advantages, and clinical translation potential.^12^ Furthermore, large‐scale high quality ex vivo isolation of MSCs is hampered by the low prevalence in human tissues and suboptimal in vitro expansion protocols, which are unable to maintain MSC essential properties required for therapeutic applications.

MSCs in vitro expansion for therapeutic applications might impact proliferative rate, homing molecules, genetic stability, transcriptional processes, multipotency, transformation, and senescence of isolated MSCs,^13^, ^14^ rising critical biosafety issues.^15^ In this context, the optimization of culture conditions (medium, serum, supplements, substrates) to preserve MSC phenotype, homogeneity, fate, and better mimic their natural microenvironment (ie, the niche) during expansion, should be integrated with processes allowing large‐scale production of quality MSC, to be used in both preclinical and clinical studies.

Isolation and ex vivo expansion of MSCs are also crucial steps to ensure adequate material for potential clinical application. Commonly, MSC isolation exploits their plastic‐adherence ability, which allows quite easy cell recovery and grow in a defined culture medium; however, this protocol implies low homogeneity of freshly‐isolated cells, which increases during long‐term expansion in vitro, necessarily combined with maintenance of differentiation ability, essential to make MSC reliable candidate for medical biotechnology.

Alternatively, the separation of MSCs could be performed by mechanical or enzymatic approaches although with higher impact on biological properties of the isolated cells.^16^

Therefore, standardized separation techniques, allowing MSC purity or optimal enrichment, could significantly improve the reliability of results from in vitro and in vivo experimental approaches as well as clinical trials.

### 1.2 Defining criteria: Properties and markers

The key step to verify MSC identity, following plastic adherence, is the assessment of cell immune phenotyping and multipotency.

Indeed, in the absence of unique and common markers, minimal criteria for MSC definition have been formulated by the International Society for Cellular Therapy (ISCT): (a) plastic adherence under the standard culture conditions; (b) positivity for CD73, CD90, and CD105 cell surface markers, coupled with the absence of the endothelial and hematopoietic stem cell proteins CD14, CD19 CD34, CD45, CD79α, and HLA‐DR; (c) differentiation into osteoblasts, adipocytes, and chondrocytes in vitro.^17^ Although MSCs from different sources share the minimum standard criteria set by ISCT, many studies suggest that each tissue of origin has a different MSC content, and influences in vitro MSC features, such as the proliferative rate, the differentiation potential, and the marker expression profile (Table 1).^18^, ^19^

On the contrary, the in vivo molecular signature of MSCs is still undefined and scantly investigated,^20^ as well as the exact matching between their in vitro and actual native behavior in vivo.^21^ In this context, CD271^+^/CD140a^−^ MSCs have been proposed to fulfill stringent stem cell criteria of self‐renewal and multipotency, also in vivo.^22^

STRO‐1 marker, not included in ISCT criteria, is used to immune‐select fresh BM‐MSCs, although it decreases during in vitro expansion of the culture^23^ and it is not univocally present in MSCs, but it is also expressed by endothelial cells.^24^ Also CD146/MCAM, which can be used to enrich BM‐MSCs cultures, has been proposed for isolation of functionally homogenous MSC populations.^25^

This set of markers is not exclusively expressed by MSCs, some of them are indeed shared (ie, with fibroblast) and highly modulated during long‐term culture. The lack of unambiguous markers may affect MSC functionality and efficacy between various studies and trials as described below in the following paragraphs.

Besides the expression of surface markers, MSCs are characterized by functional properties such as self‐renewal and multilineage differentiation. MSC cultures undergo chrondrogenic, osteogenic and adipogenic lineage differentiation when standard protocols are used, but other specific differentiation media can promote smooth muscle and striated muscle phenotype^26^ and expression of cardiac and liver genes.^27^ Typically, in vitro, BM‐MSCs can differentiate into adipocytes, osteoblasts, or chondroblasts^28^ after exposition to specific stimulating factors (ie, dexamethasone for osteogenic differentiation; TGF‐β and high cell‐density for chondrocytes; and dexamethasone, insulin, isobutyl methyl xanthine, and indomethacin for adipocytes) for 1 to 3 weeks. Confirmation of trilineage differentiation is a valuable basis to verify MSC identity from different tissue origin which, however, may diverge for differentiation potential^29^ and show a species‐dependent plasticity.^30^ Moreover, cellular heterogeneity of the cultures, likely including also committed cells or progenitors, makes differentiation capacity not identical among MSC cultures, limiting multiple lineage differentiation.^31^ Therefore, the translatability of in vitro biological features into in vivo effects may not be as clear‐cut as could be expected.

A better definition of surface markers and global molecular signatures of MSCs will help to determine and predict their effective multipotency.

However, because MSCs display high plasticity and can form any cell type, other studies indicate that alternative in vitro conditions can trigger MSC transdifferentiation into multiple cell lineages, such as cardiac,^32^ muscle,^33^ endothelial cells,^34^ astroglia,^35^ pancreatic islet cells^36^ renal tubular epithelium,^37^ keratinocytes,^38^ and hepatocytes.^39^ However, reliability and significance of MSC differentiation into mesodermal and nonmesodermal lineages is still highly controversial, and there is no evidence that occurs in vivo.

### 1.3 Overview of MSC functional properties

MSC peculiar biological properties have been exploited for stem cell‐based therapeutic approaches both in preclinical and clinical studies. The favorable properties such as extensive proliferation, multipotency, ability to migrate and home to the site of injury or inflammation, and immune‐modulation boost MSCs use in regenerative medicine, and as vehicle for gene and drug delivery into diseased areas. As therapeutic agents, MSC can act directly by cell‐cell interactions or indirectly, by secreting factors (growth factors, chemokines, cytokines, exosomes), which modulate cell and tissue functions.

In vivo, MSCs display complex biological features and behavior highly dependent on their genetic profile and the surrounding microenvironment of the anatomic sites in which they reside that are formed by different cell types and extracellular matrix (ECM) composition. Therefore, both humoral and cell‐cell signaling mechanisms control MSC growth, mobilization, and differentiation.

MSC ability to migrate and accumulate into inflamed, ischemic, or injured tissues, as well as in tumors, contributes to tissue repair or regeneration. MSC homing has been definite as the active or passive arrest within vasculature followed by transmigration across endothelium.^40^ Regenerative medicine mainly exploits MSC differentiation to support heart and lung tissue injury repair; nevertheless, the therapeutic efficacy is often debated due to the incomplete cell characterization, and the inconsistent in vivo viability, distribution and engraftment. Preferential MSC homing to damaged/inflamed sites in vivo is steered through a gradient of chemokines released from the injured sites, using a leukocyte‐like multistep extravasation, following a cascade process by rolling, activation, firm adhesion, and transmigration.^41^ This multistep mechanism involves several molecules (eg, CXCR4/7, VLA‐4, ICAM‐1, CD44, matrix metalloproteinases [MMPs]) (for a review, see Reference 42). Homing is finalized by migration steps by which MSCs reach, through the interstitium, the injured areas, further favored by other chemotactic cues released by both damaged tissues and immune cells, including growth factors (vascular endothelial growth factor [VEGF], platelet‐derived growth factor [PDGF], insulin‐like growth factor 1 [IGF‐1], fibroblast growth factor [FGF]), cytokines and chemokines (CXCL12, CXCL8, CCL5, etc.).

In vivo, besides local factors (ie, chemokine gradient), this process depends also on the site of infusion, which implies the requirement of bypassing systemic vascular barriers to reach the target tissue. In fact, MSCs can be dispensed either by site‐specific or systemic administration, through intravenous, intraperitoneal, or intra‐arterial infusion. After intravenous inoculation, the vast majority of MSCs is rapidly trapped within the capillary beds in the lungs, due to MSC size and volume, which are bigger than lymphocytes, a phenomenon termed “first‐pass” effect. Intra‐arterial infusion bypasses the “first‐pass” effect, allowing MSCs to spread peripheral tissues before to reach lungs.^43^ Of note, endogenous BM‐MSCs are smaller in size than ex vivo in vitro‐expanded MSCs.^44^ Conversely, MSC tropism seems not be limited by the blood‐brain‐barrier (BBB), formed by cellular interaction between astrocytes, pericytes, neurons, and microvascular endothelial cells.^45^

However, the molecular mechanisms responsible for MSC brain homing have not yet entirely elucidated. Several in vivo studies demonstrate MSC presence in xenograft glioma models, after either direct or systemic inoculation. In support to MSC ability to reach gliomas, several imaging methods were developed that allow to visualize and track MSC migration in vivo, from single cell level to whole body. Lipophilic fluorescent vital dye labeled MSCs, injected into the carotid artery or in the opposite cerebral hemisphere of mice bearing human glioma intracranial xenografts, were visualized through immunohistochemistry analysis of brain tissues at single cell level.^46^ Ex vivo histological detection of MSCs in the target tissues is the most common method used exploiting a large number of fluorescent vital dyes.^47^, ^48^, ^49^ Alongside these techniques, whole body imaging methods have been developed and allowed to study the kinetics and the distribution of MSCs in live animals. The main and most used are bioluminescent optical imaging (BLI), magnetic resonance imaging (MRI), and nuclear imaging techniques.^50^, ^51^, ^52^, ^53^, ^54^, ^55^ For a in‐depth review on MSC homing imaging, see Reference 43. In a syngeneic rat model, luciferase charged MSCs, injected in the right common carotid artery, were tracked by in vivo BLI into right frontal lobe, co‐localizing with glioma cells. Similar results were observed using a fluorescent vital dye, after injection into the same site. However, no homing was detected when MSCs are intravenously injected.^51^

Homing of MSCs to human brain tumors was studied comparing three main imaging methods. Fluorescent vital dye, luciferase, or ferumoxide charged MSCs were analyzed by BLI or MRI or immunofluorescence for homing U87 glioma mice xenografts. Contralateral migration of forebrain inoculated MSCs was detected with all three imaging systems, pointing out the useful application of all the techniques and in particular of MRI to increase imaging resolution and estimate real‐time migration.^53^ Other technical studies validate the use of gold‐coated nanoparticles or ferritin heavy chain expressing MSCs as marker to track by MRI.^50^, ^54^ Migration of AT‐MSCs to target brain tumor‐initiating cells was studied by in vivo BLI analysis using fluorescent magnetic nanoparticles.^52^, ^56^ Hsu et al demonstrated that hypoxia‐preconditioned placental‐derived MSCs (p‐MSCs) pass BBB and reach intracranial U87‐GBM stem cells in mice. They monitored tumor homing of intravenously injected p‐MSCs charged with PEG‐SPIO nanoparticles through T2‐weighted MRI, a real‐time and noninvasive imaging method. Nevertheless, most of intravenous injected p‐MSCs were trapped in the lungs.^52^ Lung entrapment and cell administration remains the main issues interfering with MSC homing and consequently MSC drug delivery, thus approaches to improve these processes (ie, reduction of cell diameters; increase capillary permeability; use of microparticles charged with MSC secretoma; use MSC‐derived EVs or exosomes) are important challenges to deal with.

A mutual influence exists between migratory and immunomodulatory properties since immune‐related factors released by MSCs regulate immune response and simultaneously MSCs are affected by paracrine modulation of immune cells (T and B lymphocytes, NK cells). This aspect increased the interest about MSC application in immune diseases, and currently their paracrine modulation of tissue cell functioning is gaining a broader perspective on MSC‐based therapy scene rather than cell replacement approaches.

MSCs exert immunomodulatory activity on both the innate and adaptive immune systems. Direct or paracrine interaction between MSC and immune cells can impair immune activity via PDL‐1 and Fas‐L, and through TGFβ, HGF, and PGE_2_, respectively. Immunosuppressive effects are related to the inhibition of T‐cell proliferation and induction of Tregs,^57^, ^58^ promoting macrophages transformation from M1 to M2 anti‐inflammatory phenotype,^59^ or contributing to immune homeostasis. MSCs also modulate maturation and functions of dendritic,^60^ B,^61^ and NK cells.^62^ However, as for other properties of MSCs, also observations on the immunosuppressive effects mainly derive from preclinical models, therefore, the clinical translation, showing divergent data of efficacy, is far from be definite.^63^

Overall, the biological properties of MSCs evidence a perspective role in oncology, based on their innate tumor tropism and release of relevant factors to modulate tumor microenvironment (TME), also considering genetically modified and loaded MSCs able to transport and deliver therapeutics to cancer sites. In this context, the oncogenic risk and pro‐metastatic effects represent crucial limitation factors for clinical applications.^64^, ^65^

## 2 MSCs IN THE TME: THE UNICITY OF GBM

MSCs display stem properties that support their self‐renewal and regenerative functions and simultaneously represent a helpful component for survival and proliferation of other cell types and stem cells within their specialized microenvironment, the stem cell niche.

Human BM‐MSCs reside in specific niches including nonhematopoietic cells (osteoblasts, adipocytes, endothelial cells) that sustain and regulate throughout life the hematopoietic compartment and osteoblasts, as either regenerative response or homeostasis of the stem cell pool.

Stem cell niches are almost ubiquitously distributed in adult tissues as well the presence of MSCs, particularly in perivascular niches harboring MSCs resembling pericytes, have been described in multiple organs,^66^ consistently with the ubiquitous distribution of capillary blood vessel. This localization might be due to the vascular/blood support and MSC recruitment needed during regenerative processes. However, the lack of peculiar markers to discriminate MSCs from pericytes, and their similarity to BM‐MSCs makes challenging their exact characterization, even if a common developmental derivation for perivascular MSCs could be hypothesized.

Interestingly, perivascular MSCs also reside in the human brain tissue, within neuro‐vascular niches, with endothelial cells, astrocytes, and neurons,^67^, ^68^ as well as in mouse brain, where MSCs, morphologically similar to BM‐MSCs, have been described.^69^

There is increasing evidence of the critical involvement of MSCs in the TME, and therefore in cancer development and progression. MSCs are recruited within tumors and tightly interact with the other cell types present in the TME, since tumor site is affected by a chronic state of inflammation. Thus MSC homing, fate and reprogramming is driven by cues produced by diverse resident cells composing the tumor stroma (endothelial cells, fibroblasts, pericytes, adipocytes, immune cells), cancer cells, and cancer stem cells (CSCs).^70^ CSCs are slowly dividing cells, display a highly invasive phenotype, and are considered the “root” of cancer recurrence.

MSCs exposed to tumor cell‐conditioned medium can differentiate into cancer associated fibroblasts (CAF)^71^, ^72^, ^73^, ^74^ by TGFβ1‐mediated mechanisms, promoting tumor invasion, epithelial‐mesenchymal transition (EMT), ECM modification, and cancer cell stemness, leading to tumor progression and metastasis.^75^

In particular, a back‐and‐forth crosstalk between MSCs and CSCs has been shown in several tumor types^76^, ^77^; for example, in breast cancer this interaction can transform MSC into tumor‐forming cells.^78^

MSCs have been identified in the stroma of many cancers, including human GBM, likely deriving from local sites or being recruited from BM,^79^ and represent, altogether with other nonneoplastic stromal components (endothelial cells, pericytes, immune cells, and glial cells), about 50% of GBM mass.^80^

GBM, the most common and aggressive brain primary cancer, despite surgery, radiation, and chemotherapy, is invariantly lethal. GBM represents the paradigm of the role of tumor‐initiating CSCs^81^, ^82^ in the promotion of tumor aggressiveness, cell heterogeneity, and drug resistance.^83^ All these glioblastoma stem cell (GSC) functions are tightly regulated by autocrine/paracrine activation of chemokine receptors (ie, CXCR4/CXCR7),^84^ and by TME.^85^ In detail, GSC self‐renewal is sustained by reactive tumor‐associated microglia and macrophages, astrocytes, endothelial cells and other cell types present in the niches, MSCs included, which thus contribute to tumor recurrence and therapeutic resistance.^86^, ^87^, ^88^

Interestingly, in the WHO classification, which divide GBMs in proneural, neural, mesenchymal, and classical subclasses, the majority of GBMs has a mesenchymal phenotype, identified by the incorporation of peculiar molecular signatures (IDH status, ATRX loss, H3K27M mutation, TP53 mutation, 1p/19q codeletion).^89^ The clinical behavior of mesenchymal GBMs is extremely aggressive, with resistance to radiotherapy and the poorest prognosis as compared to all the other subtypes.^89^, ^90^ In addition, also GSCs from this subgroup of GBM express mesenchymal markers, being highly positive for CD44 and BMI1, and negative for CD133.^91^, ^92^ The evolution of GBM toward the mesenchymal phenotype is pushed by several factors, including stromal and immune cells within the TME, and a selective pressure induced by radio‐chemotherapy.^93^, ^94^ In fact, current GBM therapies (radiotherapy, temozolomide [TMZ], and bevacizumab as antiangiogenic drug) impact on tumor cell behavior and promote the mesenchymal phenotype, via the activation of EMT causing the acquisition of mesenchymal‐like markers and functions, such as treatment resistance and highly invasive capacity.^95^

Therefore, MSCs and mesenchymal phenotype directly and indirectly (via TME) contribute to the striking intratumor heterogeneity of GBM cells, likely supporting high cellular plasticity, GSC survival to therapeutic agents and tumor progression. The presence of MSC‐like cells within GBM stroma (GBM‐associated MSCs [GA‐MSCs]) reveals the crucial role played by these cells in CSC proliferation and tumorigenicity.^48^, ^79^ Tumorigenic MSC‐like cells, have been identified in GBM specimens, located around vessels in the vascular niche, by the expression of mesenchymal markers (Lin‐Sca‐1, CD9, CD44, CD166) and for the differentiation potential.^48^

GA‐MSCs can be recruited either from local brain sources or from the BM.^48^, ^96^ BM‐MSCs have high affinity for GBM^97^ and enhance GSC self‐renewal and invasive potential^79^; a subset of these tumor‐supporting stem cells, identified in both low and high‐grade gliomas, was reported to be able to sustain the aggressiveness of GSCs via exosome release and its presence represents a predictive parameter of bad prognosis.^98^

However, the phenotype of GA‐MSCs and BM‐MSCs often do not completely overlaps, since, besides peripherally recruited MSCs, others GA‐MSCs may origin from CSC differentiation and display genetic patterns intermediate between these two cell populations,^79^ in term of marker phenotyping (ie, differential expression of CD90), origin (differentiation of GBM cells) or function.^99^, ^100^ Therefore, different mesenchymal populations are present within GBM mass.

Several findings suggest that MSCs are involved in angiogenesis, further contributing to the malignancy of gliomas.^96^, ^101^ The ability of GA‐MSCs to differentiate into pericytes, driving the maintenance of functional vessels essential for GBM growth, has been also described,^100^ and indeed CD105^+^ MSCs are localized around GBM arterioles.^79^ Finally, the ability of GBM‐associated endothelial cells to acquire a MSC‐like phenotype in GBM TME also contributes to chemoresistance via the activation of Wnt/β‐catenin axis and the multidrug resistance‐associated protein‐1.^102^ Furthermore, the percentage of CD105^+^/CD73^+^/CD90^+^ GA‐MSCs within tumor tissue of patients with high‐grade glioma is inversely correlated with patient's overall survival.^103^ Inflammation and hypoxia, existing in GBM, recruit MSCs in the TME concurring to modulate host immune response, promote cancer progression, or favor cell fusion. Fusion between MSC and tumor cell has been described as a possible mechanism responsible for generation of CSCs with the acquisition of both mesenchymal and stem cell‐like features.^104^ More, recently, GA‐MSCs meeting the typical mesenchymal biological profile but also expressing the stem and glial markers nestin and GFAP, PD‐L1 and secreting TGFβ, CCL2, PGE2, IL‐6, and VEGF, have been isolated. These cells, co‐cultured in vitro with peripheral blood mononuclear cells, are able to reduce Th17 lymphocytes and increase Tregs, promoting tolerogenic phenotype monocyte‐derived cells, likely contributing to the immune suppression observed in GBM.^105^

Overall, current studies highlight the complex biology of MSCs in normal and tumor tissues, whose interactions with neighboring cell types can trigger both disease‐promotion and therapeutic responses.

MSCs support maintenance of CSCs, tumor cell proliferation rate, differentiation into pro‐tumorigenic stromal cells, immune suppression, neoangiogenesis, EMT, and metastasis. Conversely, MSCs may exert antitumorigenic activity via improvement of the immune response, inhibition of angiogenesis, and activation of antiproliferative and pro‐apoptotic pathways.

The complex crosstalk between MSCs and the TME in GBM is detailed in the following paragraphs, to accurately assess the impact and mechanisms of MSCs on GBM progression.

## 3 CROSSTALK BETWEEN MSC AND GBM CELLS: DIRECT AND INDIRECT INTERACTIONS

The exact role of MSCs in GBM TME is still debated and far long to be clarified. MSCs are described alternatively as pro‐ or antitumorigenic, depending on the type and source of MSCs, the use of GBM cell lines or GSCs, and the in vitro or in vivo models that are investigated. Moreover, direct interaction using cocultures or indirect effects mediated by MSC secretome or the EVs released, have generated contrasting results that get confusion about their actual role.

To overcome the difficulties encountered in interpreting the results obtained by in vivo tumor xenografts, in vitro basic tests were developed. The simplest model consists in the study of the conditioned medium released by MSCs and its influence on GBM cell growth, survival, and in vitro migration. MSC secretoma is considered the main effector of their regenerative, tropic, trophic, angiogenic and immunomodulatory functions. However, the activity of the released substances is dependent on the tissue of origin of MSCs and the microenvironment where they are analyzed. Therefore, it is relevant to study the cross talk between MSC and GBM cell populations and in particular, how MSC secretome changes after cocultures with cancer cells.

A comparative analysis of the major studies analyzed in this review is reported in Table 2.

**TABLE 2:** In vitro and in vivo studies reporting MSC modulation of GBM cell proliferation

MSC source	GBM cell model			Study type		MSC‐GBM interaction		Study results		Reference
	Cell line	Primary culture	GSC	In vitro	In vivo	Indirect	Direct	Pro‐tumorigenic effects	Anti‐tumorigenic effects	
UCB‐MSC AD‐MSC	U87	GBM#1 GBM#12		Yes	Yes	Yes	Yes	UCB‐MSC: inhibition of cell growth	AD‐MSC: promotion of cell growth and angiogenesis, inhibition of apoptosis	Akimoto et al^123^
UC‐MSC			CSC1 CSC2 CSC3	Yes	No	Yes	Yes	Direct co‐culture inhibits cell growth	CM promotes cell proliferation	Bajetto et al^119^
Fetal BM‐MSC Fetal M‐MSC	U251 A172			Yes	No	Yes	Yes	CM: no effect	Co‐culture induces early stimulatory effects on cell proliferation, later inhibitory activity	Chistiakova & Polianskaia^128^
UCB‐MSC	U251 SNB‐19	4910 5310		Yes	Yes	No	Yes	Induction of cell apoptosis by downregulation of XIAP		Dasari et al^114^
BM‐MSC	ΔGli36	NNI32		Yes	Yes	Yes	Yes	Reduction of tumor volume and vascular density		Ho et al^120^
GA‐MSC BM‐MSC			GSC	Yes	Yes	Yes	Yes		In vitro and in vivo promotion of cell growth and stemness through the IL‐6/pg130/STAT3 pathway	Hossain^79^
BM‐MSCs UC‐MSCs			NCH421k NCH644 NIB26 NIB50	Yes	No	Yes	No	CM induces cell cycle arrest, downregulation of cyclin D1, senescence and increases TMZ sensitivity		Kolosa et al^124^
GA‐MSC	X01			Yes	Yes	Yes	Yes		Enhancement of invasiveness by HA deposition via autocrine C5a/ERK1/2/HAS2 activation	Lim et al^106^
GA‐MSC	X01			Yes	No	Yes	Yes		Pro‐invasive matrix remodeling by CCL2/JAK1 activation and actomyosin contractility	Lim et al^107^
GA‐MSC BM‐MSC	X01 TS11‐16 TS09‐03		GSC11	Yes	Yes	Yes	Yes		Promotion of invasiveness through Ca5, p38/ZEB‐1 axis	Lim et al^108^
AD‐MSC	U87		U87‐CSC	Yes	No	Yes	No		No effects on cell growth, stemness and TMZ sensitivity, increase of cell migration	Onzi et al^125^
UC‐MSC BM‐MSC AD‐MSC	U87			Yes	Yes	Yes	Yes		Increase of cell proliferation. Cell–cell contact enhances proliferative and invasive cell behavior and tumor development in vivo	Rodini et al^126^
BM‐MSC	U87 U373			Yes	No	Yes	Yes	Inhibition of cell proliferation and migration; syncytium and fusion formation		Schichor et al^113^
BM‐MSC			SU3	Yes	Yes	No	Yes		Cell fusion contributes to GBM neovascularization	Sun et al^112^
BM‐MSC			SU3	Yes	Yes	No	Yes		In vivo, cell fusion enhances angiogenesis	Sun et al^49^
UCB‐MSC	U251	5310		Yes	Yes	No	Yes	Induction of cell cycle arrest through cyclin D1 downregulation		Velpula et al^115^
UCB‐MSC	U87 U251	4910 5310	U87‐CSC U251‐CSC 4910‐CSC 5310‐CSC	Yes	Yes	No	Yes	Reduction of cell invasion and growth; induction of MET; reduction CSC phenotype		Velpula et al^118^
UC‐MSC	U251 SNB‐19			Yes	Yes	Yes	Yes		Increase of cell proliferation, migration, no effects on TMZ sensitivity	Vieira de Castro et al^127^
AD‐MSCs UC‐MSCs	U251			Yes	No	Yes	No	Inhibition of cell growth by inducing apoptosis and differentiation		Yang et al^122^

Abbreviations: AD‐MSC, adipose MSC; BM‐MSC, bone marrow MSC; CM, conditioned medium; CSC, cancer stem cells; ECM, extracellular matrix; GA‐MSC, glioma associated MSC; GSC, glioblastoma stem cells; HA, hyaluronic acid; ISCT, International Society for Cellular Therapy; MET, mesenchymal epithelial transition; M‐MSC, muscle MSC; UC‐MSC, umbilical cord MSC; UCB‐MSC, umbilical cord blood MSC; TMZ, temozolomide; XIAP, X‐linked inhibitor of apoptosis protein.

### 3.1 Direct MSC‐GBM cell‐to‐cell interaction

The crosstalk between MSCs and GBM cells, besides protein and vesicular component exchange, is strictly dependent on and sustained by their close contact that is established in the TME.^93^ GA‐MSCs, as key components of GBM stroma, promote proliferation, stemness, and tumorigenicity of GSCs, through the release of IL‐6 and the activation STAT3 in cancer cells. Most GA‐MSCs isolated from human GBM tissues are genetically distinct from the GSC paired, although some of them may derive from GSC differentiation.^79^

In a murine syngeneic GBM model, GL261 cells, inoculated into the brain, recruit MSCs from host to the tumor site, and the presence of MSC infiltration correlates with tumor progression. GA‐MSCs show a definite mesenchymal phenotype (Sca‐1^+^/CD9^+^/CD44^+^CD166^+^) that is also expressed by GL261 cells.^48^

Su‐Jae Sun's group focused its studies on the role of GA‐MSCs in the remodeling of ECM favoring the invasion of GBM cells, through hyaluronic acid (HA) deposit in the microenvironment. This occurs via an autocrine mechanism regulated by C5a secretion, which, in turn, activates HAS2 receptor and ERK1/2 MAPK to cause HA release.^106^ GA‐MSC modifications of GBM microenvironment are also dependent on CCL2 release, which mediates the activation of JAK1 to regulate actin‐myosin contractility and TME and tissue mechanical stiffness needed to promote the motility of tumor cells. Therefore, GA‐MSC contribute to ECM remodeling modifying GBM stiffness similarly to what CAFs do in carcinomas.^107^ Recently, the same authors reported that C5a released by GA‐MSCs increases ZEB1 levels in GBM cells through the activation of C5aR1 and p38 MAPK signaling, promoting the invasiveness of GBM without modifying growth rate in vitro. C5a secretion is increased by coculture with GBM cells pointing out a cross talk between GA‐MSC and GBM. Conversely, BM‐MSC co‐inoculated with GBM cell neither release C5a nor modify tumorigenesis and mice survival, revealing an intrinsic difference between GA‐ and BM‐MSC origin although both cells types express the same mesenchymal markers and differentiation ability.^108^

It is generally accepted that the serum‐free culturing conditions preserve GSC tumorigenicity, while the presence of fetal serum in the culture medium allows the development of nontumorigenic cells causing the depletion of nonadherent tumorigenic population.^109^, ^110^ However, by interaction with MSCs, GSCs are able to retain their tumorigenic ability. In fact, it has been reported that in a mixed cell culture, adherent mesenchymal‐like cells may act as feeder layer to allow nonadherent GSCs to grow also in the presence of serum, retaining the tumor stem‐like features.^111^

The influence of BM‐SCs (labeled with green fluorescent protein [GFP]) on GBM development was also addressed analyzing their modulation in coculture with GBM progenitor cells (labeled with red fluorescent protein [RFP]). These in vitro culture conditions induced the fusion of the two populations (identified as GFP^+^/RFP^+^ cells) that, when injected into mice, transdifferentiated causing the formation of a solid and vascularized tumor in vivo.^112^ Thus it was proposed that cell fusion may represent a major driving factor for GBM neovascularization. This observation was then confirmed in a more recent paper by the same authors. The injection of RFP‐GBM cells into the caudate nucleus of GFP‐mice allowed the isolation of GFP^+^/RFP^+^ cells, representing fused cells. GBM‐BM‐MSCs fused cells showed enhanced endothelial marker expression (CD31, CD34, VE‐Cadherin), in addition to stem cell markers, and enhanced angiogenic and tumorigenic ability, as compared to parental glioma cells.^49^ Conversely, other studies using cocultures of BM‐MSCs and U87 and U373 glioma cells reported a reduction of proliferation of both populations due to the generation of a syncytium between mesenchymal and glioma cells, mainly mediated by gap‐junctions. However, although the fusion events are more manifest in cells grown as spheroids than in monolayers, enhanced migration of glioma cells out of spheroids in cocultures was observed as compared with monocultures of either BM‐MSC or GBM spheroids.^113^

Rao's group published a series of studies supporting the ability of human UCB‐MSCs to reduce GBM growth both in vitro and in vivo. Authors proved that UC‐MSCs exert their antitumor action on glioma cell lines inducing apoptosis and cell growth arrest, via downregulation of the antiapoptotic protein XIAP^114^ and the cell cycle regulatory proteins, cyclin D1 and CDK 4, leading to cell death.^115^ All these effects were confirmed in nude mice in which the sizes of intracranial xenografted tumor was reduced of about 1/3 after UCB‐MSCs injection. (It has to be noted, however, that PLoS One Editors^116^ retract a previous study from these authors on the same topic^117^ due to concerns questioning the reliability of the reported results). UCB‐MSCs cocultures were also reported to inhibit GSC growth decreasing SOX2 and Twist 1 expression levels, in addition to other EMT markers. Likewise, in vivo UCB‐MSCs reduced established glioma grafted in nude mice, modulating SOX2 and Twist 1.^118^

In our lab, we assessed the direct interference of UC‐MSCs on GSC cultures isolated from both human neural and mesenchymal GBMs. The study showed that the coculture of 3D‐spheroids obtained from each cell type and differentially labeled, favors reciprocal tropism, with the fusions of the spheroids after 4 days in culture. In particular, DiI‐labeled UC‐MSCs migrated into GFP‐expressing GSC spheroids, as well as invasion of the red UC‐MSCs spheroid by green GSCs was evident.^119^ To determine whether this direct interaction between UC‐MSCs and GSCs might interfere with tumor growth, GSC proliferation was evaluated using CFDA‐SE dye dilution assay by flow cytometry, that allows tracking cell division as sequential halving of initial fluorescence in daughter cells, and by measuring Ki‐67 labeling by cytoimmunofluorescence analysis, using cocultures of differentially labeled cells. In both assays, we showed that cell‐cell contact reduced proliferation of both UC‐MSCs and GSCs, without causing cell death.^119^ A similar approach was also used by Ho and coll., which showed that BM‐MSCs reduce proliferation of GBM cells using either cell lines (ΔGli36) or primary cultures from biopsies. Moreover, they confirmed these results in vivo, xenografting subcutaneously both cell types in equal ratio, and observed that besides smaller volumes, tumors originated in the presence of BM‐MSCs were visibly less vascularized. Conversely, tumor growth was not affected by co‐transplant with immortalized normal human astrocytes.^120^

Thus, although a direct interference of MSCs on GBM cell growth was reported by different studies causing the activation of cytostatic pathways, cell fusion also favored cell migration and proangiogenic transdifferentiation, highlighting the potential complexity of the use of MSCs as therapeutic agents for GBM.

### 3.2 Indirect MSC‐GBM cell‐to‐cell interaction: Paracrine effects of released cyto/chemokines

Besides effects mediated by direct cell‐to‐cell interactions or acting as stem cells, in which differentiation ability allows to replace damaged cells, MSCs can exert physiological (and pharmacological) activities through paracrine mechanisms. However, in many cell systems, this latter mechanism is absolutely predominant and MSC stem cell nature has been challenged. Thus in the recent years several authors modified the definition of MSC into “mesenchymal stromal cells” to underline that in vivo MSCs exert their activity mainly through the secretory function rather than through the differentiation ability, as expected by “real” stem cells.^121^ MSC secretome is composed of a large population of secreted proteins and peptides and a vesicular fraction, being the latter composed by macrovesicles and exosomes, able to vehicle genetic material. The most common and relevant molecules present in the MSC secretome are cytokines, chemokines and growth factors. For example, in our study, a strong production of molecules involved in inflammation, angiogenesis, cell migration and proliferation, such as IL‐8 (CXCL8), GRO‐related peptides (CXCL1, 2 and 3), ENA‐78 (CXCL5), MCP‐1 (CCL2), and IL‐6, was observed in UC‐MSC cultures.^119^

Conditioned medium of AT‐ or UC‐derived MSCs was shown to reduce cell proliferation of U251 glioma cell line, as well as in other tumor cell types. This antitumor effect is mediated by increasing apoptosis and stimulating in vitro differentiation of glioma cells.^122^

Nevertheless, it has been reported that MSCs from different sources can have opposing effect when co‐cultured with GBM cells, derived from either primary culture or established cell lines. UC‐MSCs inhibit GBM cell growth and cause apoptosis via activation of tumor necrosis factor‐related apoptosis‐inducing ligand (TRAIL), whereas AT‐MSCs promote cell growth. However, the conditioned medium collected from UCB‐MSCs did not affect GBM cell growth, suggesting a role for a direct interaction among cells, while that from AT‐MSCs supported the proliferation of GBM cells, being the cells enriched in the mRNAs controlling angiogenic factors (VEGF, angiopoietin 1, PDGF, IGF‐1, and CXCL12). Subcutaneous co‐injection of cells from a GBM primary culture with UCB‐MSCs in Matrigel confirmed the ability of UCB cells to reduce tumor growth, whereas AT‐MSCs generate highly vascularized tumors. Thus the authors propose that these differences must be considered when choosing a stem cell source for safety in clinical application.^123^

Paracrine activities of conditioned medium from both BM‐ or UC‐derived MSCs was also reported to cause cell cycle arrest and senescence, in GSCs and GBM primary cultures by reducing cyclin D1, without inducing apoptosis or cell death.^124^ MSC secretome promotes differentiation of GSCs, downregulating the stemness markers SOX2 and Notch‐1, upregulating the glial markers GFAP and vimentin, and resulting in increased sensitivity to TMZ and 5‐fluorouracil (5‐FU).^124^ MSC‐dependent paracrine antiangiogenic effects were also proposed in light of the impairment of the endothelial progenitor cell capacity to form endothelial tubes in the presence of conditioned media derived from MSC/glioma coculture, an effect that was related to reduced PDGF and IL‐1 release.^120^

In contrast, a subsequent study reported that AT‐MSCs conditioned medium does not influence the proliferation rate and TMZ response in the human GBM U87 cell line, but increases cell migration, without modifying CSC markers expression or spherogenic ability.^125^

In a more recent paper, Rodini et al describe TGFβ as the main cytokine released by UC‐MSCs able to stimulate the growth in U87 cells, since its silencing abolishes the proliferative cue of UC‐MSC conditioned medium. When co‐injected with U87 in nude mice, UC‐MSCs increase tumor formation, although in vivo this effect is mediated through a TGFβ‐independent mechanism. Thus the Authors explored the changes occurring in the conditioned medium of MSCs co‐cultured with U87: regardless from TGFβ expression, 126 proteins with 10 new expressed proteins were detected, some of which exclusively found after cell‐to‐cell contact, such as profilin2 (PFN2), which regulates actin polymerization, cortactin, a cytoskeletal protein overexpressed in invasive tumor cells, and ezrin, an invasion‐associated protein, indicating that UC‐MSC may exert pro‐tumorigenic effects when in close contact with tumor cells.^126^

Vieira de Castro et al reported that UC‐MSC secretome increases viability, migration and proliferation of GBM cell lines in vitro, without changing sensitivity to TMZ.^127^ Likewise, in vivo, using the chick chorioallantoic membrane model, UC‐MSC conditioned medium increases tumor growth and vessel density. Proteomic analysis of secretome identified about 700 proteins with significant enrichment in Wnt, PDGF and VEGF, whose signaling pathways are often dysregulated in cancer. Other interesting proteins released by UC‐MSCs included the chemokine CCL2, semaphorin‐7A, periostin, and IL‐6.^127^ Chistiakova and Polianskaia characterized the paracrine activity of the conditioned medium of fetal MSCs, from both BM and muscle origin, in different spatial and temporal conditions. No molecule presents in the secretome of MSCs affected GBM cell growth, whereas the picture changed after direct coculture: the crosstalk between U251‐MG glioma cell line and fetal MSCs impair cell growth in the early period of co‐cultivation, which turns into cell proliferation after long time of coculture.^128^

A strong relevant effect of the paracrine crosstalk between mesenchymal and glioma cells arises also from our studies. Factors released by UC‐MSCs increase GSC proliferation and migration, through the activation of ERK1/2 and Akt signaling pathways, upon CXCR2 activation, since it was reverted by a selective receptor inhibitor. Interestingly among the cytokines released at the highest levels by UC‐MSCs, CXCL1, 2, 3 and 5 are CXCR2 ligands. This suggested that the activation of this ligand‐receptor pathway may represent a major determinant of the paracrine modulation of MSC on GSC proliferation.^119^

### 3.3 Indirect MSC‐GBM cell‐to‐cell interaction: EVs and miRNAs

#### 3.3.1 *Extracellular vesicles*

EVs are other important mediators of cell‐to‐cell communication. In particular, many biological effects of MSC‐mediated by paracrine signals, in different physiological and/or pathological conditions, involve the release of EVs.^129^, ^130^, ^131^, ^132^ EVs are composed by a heterogeneous cell‐released particle population, delimited by a plasma membrane‐derived lipid bilayer, which contain both transmembrane proteins and cytoplasmic components.^133^ EV classification includes, as main components, exosomes and microvesicles (MVs) that differ in size, origin and antigenic composition.^133^ Different particle populations can be identified in MSC culture supernatants. In particular, with “small EVs” (sEVs) are defined particles characterized by a diameter ranging about 50 to 200 nm. The commonly used term “exosome” often refers to a specific typology of sEV, generated from the endosomal system, at odds with “ectosomes” (also named as MVs or microparticles) that bud from cell membrane.^133^, ^134^ In detail, intraluminal vesicles are released from cytosol as exosomes when multivesicular bodies fuse with the plasma membrane, thus the identification of sEVs as “exosomes” requires evidence for their origin from endosomal structures, and cannot only be based on size.^135^ For this reason, in a therapeutic perspective, the separation and characterization of a pure MSC‐exosome preparation requires the removal of the numerous vesicle populations of nonendosomal origin that is extremely challenging to fulfill at this time, and to obtain a pure population is without clinical relevance. Thus the term “sEV” should be adopted when these particles are tested for therapeutic purposes, being agnostic to the site of subcellular origin.^135^ This definition also accomplishes recent recommendations from the Minimal Information for Studies of Extracellular Vesicles 2018 guidelines regarding EV classification, in which physical characteristics or isolation method should be preferred to actual origin (ie, if particles show the ability to pass through a 0.22‐μm filter, are pelleted at 100 000*g*, or are smaller than 200 nm in diameter can be defined sEVs), unless it is established a defined subcellular origin, for example via the detection of specific markers.^136^ In this context, irrespectively from biological meanings, a practical classification could include: sEVs (diameter range 30‐150 nm), which also comprise exosomes, but not exclusively^137^; MVs (diameter range 50‐1000 nm), derived by outward invaginations of cell membrane^138^, ^139^; and apoptotic bodies (diameter range 50‐5000 nm), which represent fragments of apoptotic cells.^140^ Besides a certain degree of confusion in particle definition existing in the studies trying to develop a therapeutic approach using MSC‐derived EVs, a further complication also derives from the potential different activity according with the tissue origin of MSCs (see below).

Thus a rather complex scenario is currently present in literature when approaching the biological role of EVs in intercellular communications and, even more seriously, when considered for potential clinical application.^141^ Indeed, while the therapeutic application of MSC‐derived EVs seems to be simpler than the direct use of whole MSC (ie, as far as production, manipulation and storage, including their engineering to transport pharmacological active molecules, which extend their application fields), several standardization issues are still to be solved to obtain a reliable therapeutic use of these particles.^135^, ^141^

#### 3.3.2 *Therapeutic use of MSC EVs for tumor treatment*

**FIGURE 1:**
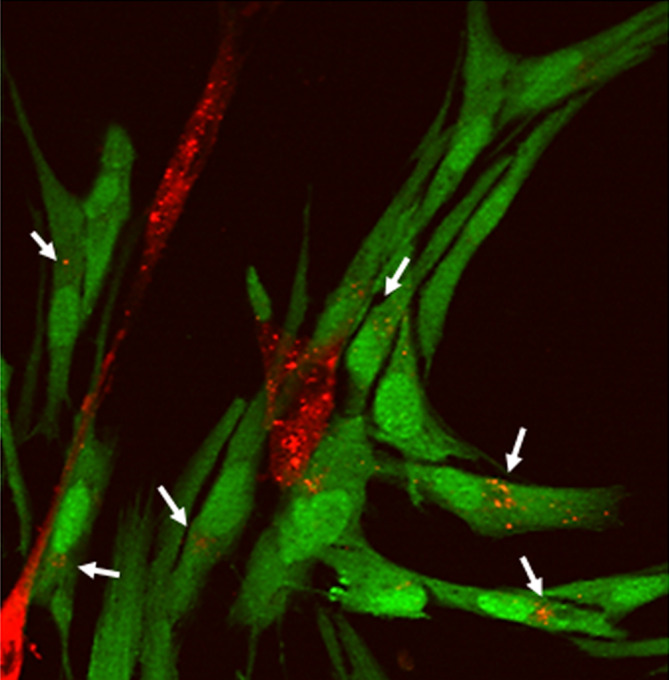
Cell‐cell interaction events in 2D monolayer cocultures of UC‐MSCs and GSCs mediated by the release of EVs by UC‐MSCs. Confocal fluorescence image of Dil‐stained UC‐MSC (red) cocultured with GSCs transfected with GFP (green): orange vesicles are found in some GSCs (white arrows)

EVs may transfer transcripts from MSCs to GBM cells, or vice versa, resulting in reprogramming the respective phenotype. For example, the transfer of transcript from MSCs to GSCs may activate migratory or self‐repair abilities. Figure 1 is a representative picture of the intercommunication between MSCs and GSCs via EVs, showing the transfer of red‐stained UC‐MSC EVs to green‐stained GSCs after coculture.

The first evidence of the potential therapeutic role of MSC‐derived EVs occurred in trials using whole MSCs to induce immunosuppression in Crohn and graft‐vs‐host diseases. However, also in the presence of beneficial effects for the patients, MSCs were not proved to persist after administration or directly contribute to the observed activity. Thus a paracrine activity, including the production of EVs, was assumed as mechanism of their therapeutic activity.^142^ This hypothesis was confirmed in some models of ischemia‐reperfusion injury by injection of MSCs conditioned medium, which showed the same efficiency of the administration of the whole cells.^143^

Following this evidence, the development of MSC EVs as innovative drugs was extended to many pathological conditions including tumors. To date, the main approach tested is the use of MSC EVs as vehicle for cytotoxic drugs or for molecules with potential antiproliferative activity (eg, miRNAs).

However, in agreement with the modulatory effects on cell proliferation observed by the direct MSC‐GSC interaction,^119^ a direct modulation of GBM cell functioning by interaction with EVs was also reported.^144^ Glioma cells in culture are able to uptake sEVs (particle analysis was reported to contain a predominant population with a diameter <150 nm) into the cytosol (measured by both confocal fluorescence microscopy and FACS). Using U87 glioma cell line, it was shown that tumor cells treated with these particles were highly modified in their proliferation rate.^144^ However, in this study, a surprising differential effect was observed using EVs isolated from MSCs of different sources. BM‐ and UC‐MSCs‐derived particles caused a significant inhibition of glioma cell proliferation (up to 50% reduction of proliferation rate) associated with a high increase in apoptosis (evaluated by FACS as sub‐G1 fraction and annexin V/propidium iodide staining). Conversely, particles derived from AT‐MSCs caused a slight (but statistical significant) increase in U87 proliferation via a speeding up of the cell cycle (increase of the number of cells entering in S and G2/M phases). This evidence suggests that variability in MSC responses and their released EVs according to the tissue origin, or due to genetic, epigenetic, or environmental factors, which may modulate MSC activity, may contribute to the conflicting data reported in the literature about their ability to affect tumor growth.^145^

However, it is important to remark that MSC‐glioma cell communication via EVs is bidirectional. Thus, as it occurs for macrophages and fibroblasts, tumor cells are able to “educate” also MSCs to use their functionality as pro‐tumorigenic activity. EVs isolated from U251 glioma cells were shown to induce a tumor‐like phenotype in BM‐MSCs, which increases cell growth and invasiveness through the modulation of the synthesis of cell cycle‐related (PCNA, c‐myc) and metastasis‐related (MMP‐2 and MMP‐9) proteins.^146^ Moreover, overexpression of CSC markers (CD133 and nestin) was also observed. From a mechanistic point of view, it was proposed that the transformation of BM‐MSCs into a tumor‐like phenotype by glioma cell‐derived exosomes can be ascribed to metabolic activation of the glycolytic pathway, as demonstrated by upregulation of Glut‐1, HK‐2, and PKM‐2, leading to the induction of glucose consumption and generation of lactate and ATP. Indeed, it was shown that secreted or EV‐delivered proteins are likely microenvironment‐dependent.^147^ For example, MSCs, transiently exposed to culturing conditions modified to better model a clinically relevant microenvironment likely occurring in vivo (ie, 1% O_2_ and serum deprivation), released EVs packaged with increased levels of extracellular‐associated proteins, among which fibronectin was the most abundant. Importantly, fibronectin mediates the mitogenic activity of these EVs on SHSY5Y neuroblastoma cells. This effect was mediated through the stimulation of the release of several growth factors (VEGF, PDGF, FGF‐7, hepatocyte growth factor) by the EV‐exposed SHSY5Y cells.^148^

Thus, the glioma‐dependent MSC conditioning represents another potentially clinically relevant variable to be considered when MSCs are planned to be used as therapeutic agents.

#### 3.3.3 *Cargo analysis of MSC EVs*

To shed light on the issue of which proteins and/or nucleic acids are transmitted via MVs, several proteomic studies were performed on MSCs and MSC‐derived EVs trying to identify possible molecular components responsible for such effects.

Comparison of BM, UC, AT, and placental MSC proteomic profile identified similar expression pattern as far as several stemness‐related genes (Oct4, Sox2, etc.) but a differential immunomodulatory activity, which was shown to be more pronounced in BM‐MSCs and AT‐MSCs, likely due to the higher expression and secretion of IL‐10 and TGFβ.^149^, ^150^ Similarly, BM‐MSCs were more active than AT‐, or lung‐derived MSCs, to inhibit eosinophil infiltration, and IL‐4, IL‐13, TGFβ, and VEGF content in experimental models of asthma determining higher therapeutic effects as far as lung inflammation and fibrosis.^150^ Consequently, also MSC‐derived EVs are expected to display a different content according to tissue origin, as far as proteins (including transcription factors) and both coding and noncoding mRNAs (including miRNAs). However, it is not possible to extrapolate data from cell analysis to obtain information on the EV content. MSC exosomes contain only 2% to 5% of the total small RNAome of the original cells and are present at high levels only few miRNAs, irrespectively from the cellular content (human BM‐MSC and AT‐MSCs were analyzed).^151^

To date, beside discrepancies in the expression between cells and EVs miRNA content, different studies reported a variety of individual molecules overexpressed or downregulated in EVs. For example, in the previously cited study, miRNAs highly expressed in MSC EVs, in comparison to the parental cells, are: (a) miR‐191, miR‐222, miR‐21, let‐7a, known to control cell proliferation; (b) miR‐222, miR‐21, let‐7f involved in angiogenesis; and (c) miR‐6087 controlling endothelial differentiation.^151^ EVs were also reported to contain regulators of transcription associated with the differentiation status of MSCs, as defined by Sox2, POU Class‐2 Homeobox 1 (POU5F1A/B), and Nanog, being, in this case, mainly tRNAs, and showing striking differences between BM‐MSC and AT‐MSCs.^151^ In a similar study, however, among a total of 413 miRNAs enriched in EVs as compared to parental AT‐MSCs, overexpression included miR‐183, miR‐378, miR‐140‐3p, and miR‐222 whose targeted genes are mainly involved in the modulation of transcription factors such as SMAD Family Member 2 (SMAD2), POU2F1, MDM4, P53 Regulator (MDM4), and One Cut Homeobox 2 (ONECUT2).^152^

#### 3.3.4 *miRNAs*

Up or downregulation of specific miRNAs have been more and more involved in the development of several tumors.^153^ For example, increased expression of miR‐199a in tumor‐associated MSCs enhances tumorigenic properties of breast cancers.^154^ miR‐221 released from MSCs derived from gastric cancer increases tumor cell proliferation and migration.^155^ On the other hand, miRNAs belonging to let‐7 family are downregulated in prostate cancer‐derived MSCs.^156^ In all these cases, it was shown that normalization of the miRNA expression/secretion abolished the tumor‐promoting properties of cancer‐derived MSCs, providing a proof‐of‐concept of the causal relationship between alteration of miRNA activity and the biological effects on tumor cells. Alteration of miRNA production/activity was also demonstrated in GBM. Tumor‐educated GA‐MSCs, isolated from human GBM surgical specimens, release exosomes, which highly increased GSC proliferation in vitro and tumor growth after xenografting of GSCs in immunodeficient mice.^157^ Interestingly, no effects were observed treating cells with GSC‐isolated exosomes. Analysis of the purified exosome fraction content demonstrated that while cytokines and growth factors were below detection threshold of the array used, it was enriched in several miRNAs. In particular, miR‐1587 was identified as a major mediator of exosome effects on GSCs, in part acting through the downregulation of NCOR1, a nuclear receptor corepressor with tumor‐suppressor activity.^157^ These results identify a relevant mechanism by which GA‐MSCs sustain tumor growth, and recognize the intercellular transfer of specific miRNAs (ie, miR‐1587), via exosome release as mechanism that enhance GBM aggressiveness.

Importantly, administration of exogenous miRNAs is potentially endowed with antitumor activity.^153^ In particular, due to peculiar biological features, MSCs can be engineered to deliver specific antitumor miRNAs to GBM cells, after being enveloped in exosomes and released.

Several studies addressed this issue and significant examples, but not an exhaustive analysis, here is reported. The identification of miRNAs with potential antiglioma activity started from the observation that several of them are downregulated in GBM, and particularly low levels of miR‐145 and miR‐124 are often detected.^158^ For example, UC‐MSCs can transfer exogenous miR‐124 to U87 GBM cells via exosomes, and mediates the inactivation of the target gene CDK6, decreasing the migration ability and enhancing chemosensitivity to TMZ of GBM cells.^159^ In another study, MSCs were transduced with a lentivirus vector expressing miR‐124a and showed that high levels of miR‐124a were specifically accumulated within exosomes.^160^ In vitro, GSC treatment with exosomes from transduced cells caused reduction of viability and clonogenicity. Deliver of exosomes containing miR124 to mice bearing intracranial GSC‐induced GBM resulted in long‐term survival in 50% of the animals, in which no histological evidence of tumor was present. Mechanistic studies showed that miR‐124a acts by silencing Forkhead box (FOX)A2, which renders GSCs unable to efficiently metabolize lipids, which intracellularly accumulate to reach toxic levels.

These data suggest that MSCs may act as natural biofactory for exosomes carrying miR‐124a with antitumor potential.^160^ The EVs secretion of MSCs has been exploited to deliver synthetic miR‐124 and miR‐145 mimics to impair GSC self‐renewal and migration through the inhibition of the expression of SCP‐1 and Sox2. Importantly, MSCs were able to deliver miR‐124 mimic to GBM xenografts, when intracranially administered.^161^ A different targeted miRNA is miR‐4731, previously identified as tumor suppressor, since it is downregulated in human tumors, including GBM^162^ and its overexpression can lead to the inhibition of cell proliferation and invasion.^163^ miR‐4731 was overexpressed in AD‐MSCs via lentiviral infection. In coculture, AD‐MSCs delivering miR‐4731 lead to a decreased U87 and U251 GBM cells proliferation, and cell migration and induced apoptosis.^162^

The transfection of MSCs with a plasmid containing miR‐146b, known to reduces EGFR expression in glioma cells as well as invasion, migration, and viability,^164^ lead its accumulation in exosomes. Intratumor injection of MSC‐transduced exosomes significantly reduced glioma xenograft growth in a rat model of primary brain tumor.^165^ miR‐133b was also reported as an inhibitory regulator of GBM growth targeting the Enhancer of Zeste 2 (EZH2) gene often upregulated in GBM tissues and GSCs.^166^ In different studies, MSC‐derived exosomes carrying miR‐133b, miR‐375, or miR‐584‐5p from transfected MSCs, repressed GBM cell proliferation, invasion, and migration in vitro and in vivo, through the downregulation of EZH2 and the Wnt/β‐catenin signaling pathway.^167^, ^168^, ^169^ Moreover, BM‐MSCs overexpressing TRAIL and miR‐7, injected in the tail vein of U87 GBM‐bearing mice released miR‐7‐enriched exosomes and suppressed tumor growth through a miR‐7‐dependent sensitization to TRAIL‐mediated apoptosis.^170^ Finally, it was shown that TMZ resistance in GBM U87 and T98G cell lines, as well as in GSC cultures isolated from human tumors, was associated to the overexpression of drug efflux transporter P‐glycoprotein, induced by high levels of miR‐9. Thus BM‐MSCs were induced to express anti‐miR‐9 and tested for the ability to affect GBM cell growth in vitro. These experiments showed that in coculture GBM cells recovered TMZ sensitivity, due to the transfer of the anti‐miR‐9 from BM‐MSCs to tumor cells via exosome secretion.^171^

Overall, MSC delivery of miRNA (or anti‐miRNA)‐containing exosomes could represent a valuable therapeutic methodology for GBM, although, also considering this approach, the choice of the target miRNA or the cell vehicle can induce opposite results.

## 4 THE POTENTIAL OF MSC‐BASED THERAPY FOR GBM

Tumor‐tropic capacity of MSCs gained attention also as a tool to deliver cytotoxic agents into cancer sites.^172^ Easy viral vector transduction, extensive protein production and expansion in vitro as well as their immune‐inert feature, associated, in the brain tumor context, with their ability to cross the BBB, favor MSC genetic engineering. Cytotoxic effects of MSC loaded with different anticancer drugs, were also prepared using liposomes and nanoparticles.

BM‐MSCs were loaded with paclitaxel and tested on T98G glioma cell proliferation in vitro. It was shown that the drug was incorporated in a sufficient amount released at cytotoxic effective concentrations when located in the proximity of the cancer cells, despite a 20% loss of cells due to spontaneous apoptosis.^173^

In vivo, the treatment with paclitaxel‐loaded MSCs of mice carrying GBM xenografts showed a high tropism of MSCs toward the tumor cells, and the ability to cause drug‐dependent cytotoxicity, without interfering with normal astrocyte viability.^174^ In another study, BM‐MSCs were primed with sorafenib without significant toxicity and were showed to be able to release up to 60% on the loaded drug. These modified MSCs were intranasally administered to mice xenografted with U87 glioma cells, 6 and 10 days after U87 cells. Analysis of the tumors after 1 week from the second MSC injection (17 days after U87 xenograft) showed a lower level of tumor angiogenesis when compared to unprimed MSCs or sorafenib alone, but no change of tumor volume was observed. The authors attributed this lack of activity to low protumorigenic properties observed administering unprimed MSCs, which balanced the cytotoxic effects of sorafenib.^175^

Human MSCs were also engineered to express on the plasmamembrane a single‐chain antibody directed against EGFRvIII, a genetic determinant of de novo GBMs, to increase cell selectivity and enhance treatment efficacy. EGFRvIII‐expressing orthotopic U87 gliomas were developed in vivo; mice intracranially treated with modified MSCs showed significantly improved survival compared to untreated animals or injected with control MSCs.^176^ MSCs, genetically modified to express cytokines, were reported to alter the immunosuppressive microenvironment in GBM models, as observed after intratumoral administration of MSCs co‐expressing high levels of IL‐12 and IL‐7 in mouse GBM, which reduced tumor growth and induced a broad tumor‐specific immune response.^177^ MSCs expressing adenoviral‐mediated IL‐18 also impair tumor growth in rat glioma models.^178^ UCB‐MSCs were also modified to overexpress CXCR4 to increase their tropism toward GBM cells^180^, which release its ligand CXCL12.^84^, ^179^ These modified cells showed a higher migration capacity in vitro, as compared to parental MSCs, and, more importantly, exhibited enhanced migration to tumor cells in intracranial glioma xenografts.^180^

Other MSC engineering strategies, assayed the possibility to deliver suicide protein, the virus‐based cytotoxicity, and antiangiogenic gene therapies. MSCs were transduced with the gene encoding pro‐drug‐activating enzymes (suicide proteins), such as the herpes simplex virus thymidine kinase (HSV‐TK), cytosine deaminase/5‐fluorocytosine or rabbit carboxylesterase, which subsequently converted the pro‐drug (ie, ganciclovir) in the active molecule with anticancer activity. This approach has been tested in human and rat GBM models.^181^, ^182^, ^183^, ^184^ A recent in vivo study describes the peritumoral and intratumoral homing of MSCs when injected in the contralateral hemisphere in an orthotopic GBM mouse model.^55^ Moreover, intratumoral release of HSV‐TK from transduced BM‐MSCs followed by ganciclovir administration, reduced tumor growth and prolonged mice survival. Analogously, HSV‐TK‐expressing UC‐MSCs limit U87 GBM xenograft growth, although a high UC‐MSC/HSV‐TK/U87 ratio was required to exert a maximal effect.^185^

Virus‐based therapy allows the supply, through MSCs, of genetically modified cytotoxic viruses to selectively kill cancer cells. A phase I trial study is currently recruiting patients with recurrent high‐grade glioma to test best dose and side effects of allogeneic BM‐MSCSs, loaded with the oncolytic adenovirus, dnx‐2401, administered via intra‐arterial injection (NCT03896568). Other oncolytic adenoviruses, CRADs and Delta‐24‐RGD, have been used for this strategy in preclinical and clinical studies to test the effective deliver to intracranial tumors. For example, testing different deliver means, it was shown that MSCs migrate and deliver CRAd to distant glioma cells.^186^ Similarly, intra‐arterially injected MSCs, expressing Delta24‐RGD, reach orthotopic U87 or U251 glioma xenografts, release Delta24‐RGD into the tumor, and improved survival and tumor eradication in subsets of mice.^187^

UC‐MSCs^188^ and AT‐MSCs,^189^ transduced to express the pro‐apoptotic molecule TRAIL, were able to reach the tumor and induce cytotoxic effects on both GBM cells and xenografts, without showing evidence of mesenchymal differentiation after injection. Moreover, mouse irradiation before TRAIL‐secreting UCB‐MSC injection synergistically enhanced apoptosis in both TRAIL‐responsive and TRAIL‐resistant GBM cells, due to an upregulation of death receptor‐5 and activation of caspase pathways.^190^ Patient‐derived GBM cell apoptosis has been also shown in mice xenografts using a polymeric nanoparticle nonviral transfection for TRAIL expression in AT‐MSCs.^191^

Finally, MSCs loaded with paclitaxel‐encapsulated poly(d,l‐lactide‐co‐glycolide) nanoparticles were tested in an orthotopic GBM rat model. Modified cells did not show changes as far as migration capacity, cell cycle progression, or multilineage‐differentiation potential, but displayed sustained paclitaxel release, both as free molecule and as nanoparticle encapsulated form. When contra laterally injected in the brain of C6 glioma bearing rats, nanoparticle‐loaded MSCs were identified within the tumor mass for 2 days after the injection. After 7 days from the injection, tumors of treated animals showed significant higher cell destruction and damaged areas showing signs of necrosis and apoptosis that control animals. Moreover, a prolonged rat survival was observed.^192^

The strong interest in exploiting the tumor‐homing ability of MSCs to target tumor cells is increasingly improving the genetic manipulation of these cells, to optimize their migration and effectiveness; however, the underlying mechanisms sustaining tumor tropism and antitumor activity, likely dependent on MSC origin and context‐dependent effects, are not completely understood and will require further studies.

As alternative approach, modified EVs from MSCs can be used as selective drug delivery system,^193^, ^194^ although in some circumstances a pro‐tumorigenic activity was also reported.^195^ Several advantages have been, in principle, identified in the use of EVs.^196^ In general, they are quite stable and there is the possibility to freeze them for later use. It is also possible to target them to obtain a directional cell‐specific uptake, for example by inducing the expression on the cell of origin of ligands, which can be transferred to the surface of the vesicles and bind to specific receptors on target cells. Thus, high performing exosome purification procedures were developed from WJ‐MSC to obtain pure populations, a fundamental condition to adopt these vesicles in clinics.^197^ Different approaches can be used to modify EV cargo to use them as precision medicine drug carriers, including overexpression of protein of interest in parental cells, antibody or antigen conjugation, chemical modification, or passive and active loading (co‐incubation with free drugs, sonication, electroporation, incubation with saponin, antibody binding).^193^, ^198^, ^199^ Several approaches were used in this perspective, including EV loading with miRNAs (see previous paragraph) or cytotoxic drugs (doxorubicin, paclitaxel, etc.). Hydrophobic and hydrophilic small therapeutic molecules can be incorporated into EVs, leading, after parental administration, to an improve drug targeting to tumor cells, obtain a slow time‐dependent release from the exosomes, and increase in potency and efficacy.^193^ While drug delivery, via EVs, and exosomes in particular, was tested in several other tumors^200^ there are not relevant studies on GBM. One relevant study was performed in *Danio rerio* xenotransplanted with U87 glioma cells and using brain cell exosomes loaded with doxorubicin. Exosomes injected into the common cardinal vein of the anesthetized *Danio rerio* embryos, were able to cross the BBB and enter the brain, reducing tumor growth.^201^

## 5 CONCLUSIONS AND FUTURE PERSPECTIVES

Regenerative, immunomodulatory, and tumor‐homing properties of MSCs have been exploited to repair tissue injuries, interfere with cancer, immune‐based disorder, and neurodegenerative disease development. This large use was also sustained by the relatively easiness of harvest and processing from different sources, and the abundant availability after in vitro expansion. Beyond regenerative medicine, MSC tumor tropism and nonimmunogenicity laid the groundwork for their application in oncology research. In particular, MSCs release soluble factors or EVs that acting via autocrine and paracrine mechanisms are able to modulate cancer cell survival and proliferation, migratory pathways, and induce host immunomodulation.

Despite initial enthusiasm, current literature highlights growing data inconsistencies and divergences on whether and how MSCs may promote or inhibit tumor growth in various cancers, including GBM. Several factors may contribute to contrasting preclinical observations, including MSC tissue of origin, isolation, ex vivo expansion, and culture protocols. Moreover, further complications may derive by the clinical setting, tumor type, administration route, timing, and quantity, often questioning the effective amount of cells homing to tumor site. All these crucial issues, hampering the research reliability and clinical improvement of MSC‐based therapies, should be addressed by in‐depth studies on biological and molecular properties of these cells.

**FIGURE 2:**
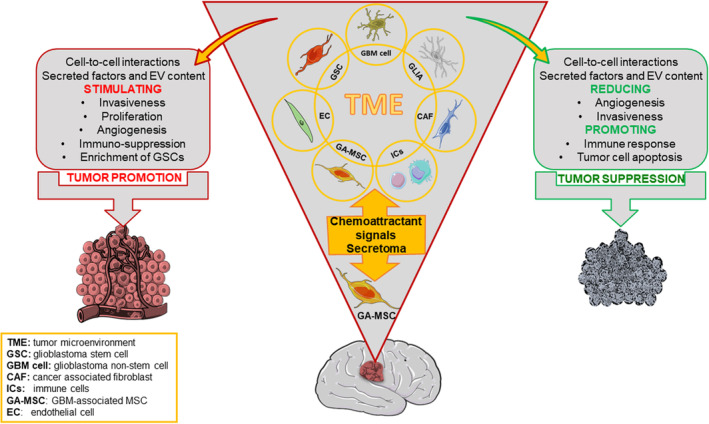
Diagrammatic representation of the interactions between mesenchymal stem cells and glioblastoma cells, also involving other nonmalignant stromal and immune cells within the tumor microenvironment. In the figure, the critical pathways that may support or impair tumor growth via a variety of mechanisms are highlighted

In the context of GBM, not only MSC effectiveness, but also the key role played by GSCs in tumor initiation, progression and drug resistance point out the need of innovative therapeutic approaches to eradicate this subpopulation. Another challenge of potential MSC‐based anticancer treatment is the complexity of the TME in which GSCs exist, where tumor cells and “normal” tumor stromal cells extensively and reciprocally impact on local milieu through secretome, cell‐cell interactions, and metabolome alterations (Figure 2).

A new frontier for MSC application is the achievement of genetic engineering‐based methodology to convert MSCs into therapeutic vehicles to graft into the tumor and produce or release engineered EVs and nanoparticles, or cytotoxic agents. These strategies supported by some initial preclinical studies, were confirmed in few human cancer studies.

The innovative MSC‐based anticancer approaches are juxtaposing with the unresolved basic questions and fixed points on MSC biology, for example, how many typologies of progenitors exist under the MSC acronym, and how can we distinguish them for their functional properties; the definition of standard protocols for MSC expansion in culture; the definition of the most reliable in vitro and in vivo GBM models based either on stable cell line or GSCs. The consolidation of this essential knowledge is the indispensable requirement to understand the crosstalk between MSC and GBM in TME.

Overall, it is clearly perceivable from the literature reported in the present review, as well as from data on the use of MSCs in different disease models and clinical trials, how hard is the comparison of the outcomes between different studies and striking controversies on the successful MSC delivery to target tissues.

Beyond oncology applications, current overwhelming clinical trials using MSCs for multiple diseases, and the simultaneous boosting of industrial interest in MSC‐therapies, not always corroborated by reliable clinical effects, warrant warning considerations.

Therefore, before accelerate clinical translation, a step back is needed to focus on the exact MSC nature, to: (a) solve the debate over the misleading MSC nomenclature and define their identity, “stem” or “stromal” identity, to indicate true stem cells or tissue‐specific committed progenitors, and to overcame the existing concept of tissue‐source equivalence; (b) consider, detect and control cell population heterogeneity which can be isolated from diverse tissues and individual patients; (c) analyze in detail, exploiting genomic and transcriptomic signatures, MSC phenotype and expression profile; (d) define and unify the MSC processing protocols, from the in vitro isolation and expansion steps to the patient treatment.

Due to the complexity of these issues and the absolute requirement for a standardization of the procedures for MSC isolation and characterization to ensure their optimal application not only in oncology, but also in other therapeutic fields, the constitution of expert panels which can carefully dissect the literature, possibly organized in subgroups according the different application of this cell therapy, are highly recommended. We believe that this approach, establishing a gold standard in the study and use of MSCs, involving basic and clinical researchers, and even biotech and pharmaceutical companies, could allow a better sharing of information resulting in a real advancement in the field.

## CONFLICT OF INTEREST

The authors declared no potential conflicts of interest.

## AUTHOR CONTRIBUTIONS

A.B., F.B., T.F.: wrote the manuscript; all authors contributed to literature searching, analysis, and critical review of the article cited, and proofed and approved the manuscript.

## Data Availability

Data sharing is not applicable to this article as no new data were created or analyzed in this study.
